# Application of Nanoliposomes Containing Nisin and Crocin in Milk

**DOI:** 10.34172/apb.2023.014

**Published:** 2021-10-26

**Authors:** Mohammad Yousefi, Seid Mahdi Jafari, Hossein Ahangari, Ali Ehsani

**Affiliations:** ^1^Department of Food Science and Technology, Tabriz University of Medical Sciences. Tabriz, Iran. Food and Beverage Safety Research Center, Urmia University of Medical Sciences.; ^2^Department of Food Materials and Process Design Engineering, Gorgan University of Agricultural Sciences and Natural Resources, Gorgan, Iran.; ^3^Department of Food Science and Technology & Nutrition Research Center, Tabriz University of Medical Sciences. Tabriz, Iran.

**Keywords:** Nanoliposome, Nisin, Crocin, Encapsulation, Milk

## Abstract

***Purpose:*** This study aimed to investigate the effects of nanoliposomes containing crocin and nisin in milk samples as a food model. Therefore, three formulations were prepared and compared, including (1) milk samples containing free nisin and crocin, (2) samples with nanoliposomes containing nisin and crocin, and (3) nisin and crocin-loaded nanoliposomes coated with chitosan.

***Methods:*** In order to find the optimum amount of both bioactives within nanoliposomes, analyses of size, polydispersity index (PDI), zeta potential, and encapsulation efficiency were accomplished. Then, the best formulated nanoliposome was evaluated and compared with a solution containing free bioactives and nanoliposomes coated with chitosan using other experiments, including antioxidant and antibacterial activities, viscosity, colorimetric and bacterial growth.

***Results:*** The best nanoliposomal system based on the factors of size, PDI, zeta potential, and encapsulation efficiency was related for the nanocarrier with 4 mg crocin, 4.5 mg nisin, and 40 mg lecithin. Based on the results obtained, both nanoliposome (a*=5.41) and chitosancoated nanoliposome (a*=5.09) solutions could significantly (*P*<0.05) reduce the redness of milk induced by free bioactives (a*=12.32). However, viscosity of milk in chitosan-coated nanoliposome solution was found to be higher (3.42 cP) than other formulations (viscosity of samples with free bioactives was 1.65 cP and viscosity of samples containing nanoliposome was 1.71 cP). In addition, chitosan-coated nanoliposomes could inhibit the growth of *Listeria monocytogenes* stronger than other samples.

***Conclusion:*** Encapsulation of nisin and crocin in nanoliposomes showed promising results for preserving food safety and quality.

## Introduction

 Production of safe and high-quality foodstuff has been almost one of the most important issues among food industries. The application of bioactive compounds such as antimicrobial and antioxidant ones in different foods has been widespread for a long time.^1,2^ One of the most dangerous threats of dairy product is *Listeria monocytogenes*. This organism is a facultative anaerobe and gram-positive bacterium that grows at refrigeration temperatures. This pathogen can be found throughout the environment, particularly in water and soil. *L. monocytogenes* may survive in foods that have a relatively high salt content and acidity and can tolerate low and high temperatures. Owing to its ubiquitous nature and the ready-to-eat traits of dairy products, *L. monocytogenes *is a pathogen of concern for processors of dairy products.^3^ It can easily contaminate milk samples from non-hygienic sampling from the cow udder or through the equipment of cow-milking.^4^ This organism manifest itself as febrile gastroenteritis, endocarditis, endophthalmitis, peritonitis, septic arthritis, spontaneous abortions etc.^5^

 Direct application of bioactive compounds in food systems usually does not have a desirable result. Low stability and solubility as well as sensitivity to environmental conditions such as heat, light, oxygen, or humidity have limited their direct usages.^6^ In addition to inherent problems associated with the bioactive ingredients, undesirable interactions of these bioactive agents with components of a food system decrease their efficiency.

 In order to decrease drawbacks of direct application of different bioactive substances in foodstuff, delivery systems for encapsulating these compounds, with purposes of protecting, decreasing unwanted reactions, and enhancing the efficiency of bioactives have been developed. Nanoliposomes are considered one of the most used delivery systems for encapsulating bioactive compounds. Nanoliposomes are mainly constituted of polar lipids such as phosphatidylethanolamine, phosphatidylcholine, or a combination of these lipids with ergosterol or cholesterol as the external bilayers and an interior aqueous phase, which allow liposomes to encapsulate both lipophilic and hydrophilic compounds.^7^ Due to their nano scale size, nanoliposomes possess a high surface area, which enables them to entrap more bioactives, be resistant against destabilization, and have a high effectiveness.^8^ Although nanoliposomes are believed to be one of the most efficient delivery systems, like other encapsulants, they have some drawbacks. The most important limitations of liposomes are their poor stability, fusion and aggregation, and low encapsulation efficacy, which induce the burst release of compounds.^9^ An applicable way to decrease the disadvantages of nanoliposomes is to coat them with hydrocolloids, which also can give nanoparticles extra antimicrobial activities, in the case of using hydrocolloids with antimicrobial properties such as chitosan. For instance, Wang et al^10^ observed that with increasing the concentration of chitosan coating from 0.25 to 4 mg/mL on nanoliposomes containing cinnamaldehyde, the antimicrobial activity of the prepared system against *Staphylococcus aureus* constantly increased. Besides, increasing the chitosan concentration meaningfully decreased the minimum inhibitory concentration (MIC) of the nanoliposomes from 200 to 12 mg/mL. Two bioactive compounds used in this study for investigating their effectiveness in milk are nisin and crocin.

 Nisin is a small peptide, which is synthesized by *Lactococcus lactis* ssp. *lactis*. This substance is frequently utilized in food industries for avoiding growth and spore germination of pathogenic gram-positive bacteria. An acceptable daily intake (ADI) of 0.13 mg/kg body weight (bw) has been previously established by Scientific Committee on Food (SCF) for nisin. Also, US Food and Drug Administration (FDA) has confirmed the safety of nisin.^11^

 Crocin is a water-soluble carotenoid in saffron. Crocin is a glycosyl ester derivative of 8,8′-diapocarotenedioic acid.^12^ Many studies have verified the antioxidant activity of saffron on reactions of free radicals, which is mostly related to crocin, having a stronger antioxidant activity compared to α-tocopherol.^13,14^ Nevertheless, the direct application of crocin is usually faced with problems such as susceptibility to lysis, sensitivity to the ambient stresses, uncontrolled functions, and unwanted interactions with food ingredients.^15^ Therefore, this study aimed to investigate the effect of liposomal encapsulation of nisin and crocin on various aspects of milk such as physicochemical and microbial properties. Given the safety of crocin, only one research has dealt with the assessment of crocin tablets safety in healthy volunteers, exhibiting a relatively safe profile at 20 mg.^16^

## Material and Methods

 All materials, including egg yolk phosphatidylcholine, chitosan, cholesterol, crocin, and nisin were purchased from Sigma Chemical Company (Sigma Chemical Company, St. Louis, MO, USA).

###  Preparation of nanoliposomes

 Ethanol injection method was used to prepare nanoliposomes according to the study of Tavakoli et al.^17^ In the first step, a determined amount of phosphatidyl choline (lecithin) was blended with 100 mg cholesterol in Erlenmeyer flasks containing 100 mL ethanol/phosphate-buffered saline (PBS, pH = 6, 0.05 M) (1:1 ratio). Then, the determined amounts of nisin (45, 60, and 75 mg) and crocin (20, 40, and 60 mg) were added to the solutions. The prepared solution was mixed vigorously using a magnetic stirrer. To completely homogenize the solution, a disperser (T 25 digital ULTRA-TURRAX) was used at 5000 RPM for 15 minutes. This solution was then diluted 1:10 (produced concentrations for nisin (4.5, 6, and 7.5 mg/100 mL) and crocin (2, 4, and 6 mg/100 mL)), then the mixing and homogenizing step was repeated once more to produce homogenized solutions. Finally, all suspensions were treated by ultrasonic homogenizer (Elma s60H, Germany) to develop nisin‐ and crocin‐loaded nanoliposomes. Sonication was carried out at the pulse mode (1 s on/1 s off). Time of sonication was adjusted to 20, 30, and 40 minutes. After the preparation step, liposomal solutions were transported to an evaporator device (KR-rotate digital, South Korea) to extract ethanol before the addition of solutions to milk. Ethanol was removed at 40°C for 20 minutes. All runs utilized in this study were obtained by Design Expert software version 11 and with the use of a 4 factor, 3 level Box Behnken Design (BBD).

###  Characterization of nanoliposomes

####  Particle size, polydispersity index, and zeta potential

 Particle size, polydispersity index (PDI), and zeta potential (ζ-potential) of nanoliposome suspensions were measured using a Zetasizer Nano-ZS (Malvern Instruments Ltd, Malvern, UK). For this purpose, nanoliposome suspensions were diluted (1:30) using deionized water in order to prevent multiple scattering impacts. All analyses were executed with at least three times replications.

####  Encapsulation efficiency

 Encapsulation efficiency of both nisin and crocin was accomplished based on the spectrophotometric method^18^ using UV-VIS spectrophotometer (Ultrospec 2000, England). The encapsulation efficiency of both nisin and crocin was separately calculated by the following equation.


$$Encapsulation\;efficency=\frac{Encapsulated\;bioactive\;in\;nanoliposomes}{Total\;bioactive\;(free\;and\;encapsulated)\;}$$

###  Fourier transform infrared spectroscopy (FTIR)

 FTIR spectroscopy was utilized to determine any interactions between bioactives functional groups and nanoliposomes. The infrared spectra were scanned by the FTIR spectrophotometer (Bruker, Vertex 70, FTIR Spectrometer), in frequency range between 4000 and 400 cm ^-1^ using the method KBr pellet with 10:100 ratio of sample to KBr.

###  Coating of nanoliposome surfaces with chitosan

 According to the results achieved from the characterizing properties and encapsulation efficiency, the optimum nanoliposome solution was selected to be mixed with chitosan. In this study, chitosan in a concentration of 1 % was blended with the optimized sample.

###  Antioxidant activity of nanoliposome solution

 DPPH method based on the study of Hwang et al^19^ was used to evaluate the antioxidant activity of samples. Briefly, 50 uL of the sample was mixed with 50 uL of 100 uM of DPPH dissolved in ethanol. Each mixture (with and without nanoliposome) was blended vigorously and stored for 30 minutes in a dark place at room temperature. The absorbance of the mixtures was recorded at 517 nm. A blank was made in a same method except that ethanol was utilized instead of the sample. The scavenging activity was determined as:


$$DPPH\;scavenging\;activity\left(\%\right)=\left(1-\left[\frac{Sample_{Abs}}{Blank_{Abs}}\right]\right)\times 100$$

In addition, the concentrations of 10 mg/mL cholesterol and 50 mg/mL lecithin were used to synthesize plain nanoliposomes and the amounts of 4 mg crocin and 4.5 mg nisin were utilized as bioactives. The selection of these amounts was based on the experiments mentioned in this study. The positive control was the solution containing free crocin and nisin.

###  Antibacterial activity of nanoliposome solution

 To determine the antibacterial impact of samples having unencapsulated nisin and crocin and nanoliposomes containing both bioactive compounds, the MIC test for *Listeria monocytogenes* was employed. In brief, a solution with 10^8^ cells of bacteria was prepared using the 0.5 McFarland solution. Samples with different concentration of both nisin and crocin were prepared in Mueller Hinton broth in 96-well microtiter plates. The bacterial solution was added to each well to obtain a final bacterial concentration of 10^7^ cfu/mL. The inoculated microplates were then incubated at 37°C for 24 hours. MIC was achieved by monitoring the optical density at 600 nm with a microplate reader (Multiskan MK3, Thermo Fisher Scientific, 252 Waltham, MA).

###  Formulation of milk enriched with nisin-and crocin-loaded nanoliposomes

 Sterilized low fat milk pertained to the Mihan food industries group was purchased. Then, 20 g of the solutions, including unencapsulated nisin and crocin and the nanoliposome solution (with and without chitosan) containing both nisin and crocin were blended with 100 mL of milk.

###  Color measurement 

 Prepared milk samples were subjected to the color measurement device (Hunter Lab colorimeter (Minolta model CR-410, Tokyo, Japan). The effect of unencapsulated and encapsulated nisin and crocin on the color of milk was determined. Color values, including, L* (lightness), a* (red/green) and b* (yellow/blue) were obtained.^20^

###  Viscosity

 Viscosity measurement of milk samples was accomplished using a Brookfield digital viscometer (model DV II + , USA), operated at 100 rpm at 25°C. Viscosity of all milk samples was reported as *cP*.

###  Bacterial growth 

 In order to examine the effect of different formulated milk samples on the growth of *L. monocytogenes* in a determined period, bacterial growth test was carried out. In this regard, a solution containing the number of 10^3^ CFU/mL of *L. monocytogenes *was prepared and added to the sterilized milk samples having the free bioactives, nanoliposome-loaded bioactives, and nanoliposome-loaded bioactives coated with chitosan. All samples were stored in a refrigerator (4 °C) for a period of 16 days. The number of colonies grown on the surface of *Listeria* selective agar (Quelab, Canada) cultured by spread plate technique was calculated on the days 0, 4, 8, 12, and 16.

## Result and Discussion

###  Particle size, polydispersity index, and zeta potential

 Particle size, PDI, and ζ-potential are key factors of nanoliposome characterization due to their direct effect on the functions of these delivery systems. Nanoliposomes with diameter less than 50 nm are unstable and have a high tendency to fuse with other nanoliposomes and settle down because of their high surface tension. On the contrary, bulky nanoliposomes with diameter greater than 200 nm are stable, but their advantages over free substances are questionable. Nanoliposomes having a moderate diameter from 50 to 200 nm have usually shown comparatively prolonged physical stability, and so they efficiently transport a determined amount of drugs or bioactive compounds to targeted cells.^21^ The advantage of nanoliposomes over microliposomes against bacteria is mostly related to their differences in terms of size. Decreasing the size of liposomes from micro to nano enhances its ability to accompany more antibacterial compounds to targeted cells due to the increased surface to volume ratio. The other reason is related to easier passive cellular absorption of nanostructures.^22^

 PDI is utilized to measure the particle size distribution. A favorable range of PDI should be lower than 0.5. Higher PDI values show that nanoliposomes have been synthesized in an inappropriate process or conditions owing to fabricating both small and large particles without a desirable homogeneity.^23^ The other important factor of nanoliposomes is the ζ-potential. This index indicates the overall charge of particles obtaining in a specific medium. The zeta potential of nanoliposome surfaces is a principal factor, demonstrating the stability of nanoliposomes. Normally, the acceptable ζ-potential is believed to be < -30 mV or > + 30 mV (both are indicated as high zeta potential), which causes a good stability and prevents the precipitation or aggregation.^8^

Table 1 shows all runs and responses obtained in this study. In this study, the range of size, PDI, and ζ-potential of formulated nanoliposomes were found to be 20 to 421 nm, 0.29 to 0.6, and -3.3 to -16.9 mV, respectively. According to the results, the size of produced nanoliposomes was strongly related to the amount of both nisin and crocin, so that increasing the amount of both bioactive compounds led to the increased size of particles. However, the amount of these bioactives did not exhibit a significant (*P* > 0.05) impact on the PDI of nanoliposomes. It has been suggested that PDI is mostly associated with ultrasonication time. A uniform and homogenous size of liposomes mainly depends on longer transmission time from the ultrasound probe, which promotes suspension mixing and, subsequently, greater homogeneity is achieved.^24^

**Table 1:** Obtained results for size, PDI, zeta potential, and encapsulation efficiency

**Crocin** **(mg/100 mL)**	**Nisin** **(mg/100 mL)**	**Lecithin** **(mg/100 mL)**	**Ultrasound time (min)**	**Size (nm)**	**PDI**	**Z potential (mV)**	**Crocin encapsulation efficiency (%)**	**Nisin encapsulation efficiency (%)**
2	6	30	30	40	0.4	-3.9	18.6	25.6
6	6	50	30	421	0.54	-15.4	25.1	46.3
4	7.5	50	30	415	0.55	-11.2	25.2	44.3
6	7.5	40	30	421	0.5	-4.2	22.2	28.4
4	6	30	40	41	0.29	-4.1	25.5	22.8
2	4.5	40	30	175	0.55	-8.2	19.3	29.2
4	6	40	30	222	0.53	-7.1	26.3	28.2
4	6	40	30	285	0.55	-6	27.4	25.6
4	4.5	30	30	20	0.4	-4.1	22.8	20.1
4	6	50	40	295	0.42	-11.3	28.5	49.1
2	6	40	40	202	0.35	-8.2	21.2	32.7
4	6	40	30	298	0.57	-6.2	27.2	22.5
4	7.5	40	20	370	0.6	-4.6	17.9	26.1
4	4.5	40	20	180	0.6	-8.5	22	28.9
4	6	40	30	295	0.5	-6.7	24.8	23.6
6	6	30	30	117	0.42	-4	23.7	24.2
2	6	40	20	318	0.56	-7.6	14.2	25.4
6	6	40	20	315	0.59	-8	24.2	28.8
6	4.5	40	30	262	0.45	-8.3	26.9	30.1
4	6	50	20	317	0.6	-16.3	20.2	48.3
2	7.5	40	30	356	0.49	-4.9	20.8	28.6
4	7.5	30	30	111	0.45	-3.4	24.2	22.1
4	6	40	30	305	0.48	-7.1	24.7	30.4
4	6	30	20	103	0.55	-3.3	18.5	20.5
4	4.5	40	40	199	0.3	-14.6	26.2	48.2
2	6	50	30	262	0.52	-14.7	19.6	42.2
4	7.5	40	40	403	0.4	-4.4	27.2	27.8
6	6	40	40	352	0.32	-8.2	28.8	34.9
4	4.5	50	30	238	0.51	-16.9	25.8	44.2

 Concerning the ζ-potential, nanoliposomes did not reach the value lower than -30 mV, thus a considerable stability was not observed between nanoliposomes. The unfavorable results of ζ-potential are probably owing to the presence of nisin. Nisin is a positively charged peptide because of the presence of amino groups within its structure. Negative charge values of nanoliposomes are typically attributed to the existence of lecithin terminal groups (phosphoric groups),^25^ however, amino functional groups of nisin have probably neutralized the negative charges induced by lecithin, leading to the production of less negatively charged nanoliposomes.^26^

###  Encapsulation efficiency

 Encapsulation or loading efficiency is the amount of encapsulated substances per amount of total added compounds, showing the percentage of substances successfully adsorbed or entrapped in nanoparticles.^27^ This index depends on many variables such as the amount of compounds added, pH, temperature, zeta potential, size of particles, and so on.^28^ The result of this study displayed a significant difference in the encapsulation of crocin ranging from 14.2% to 28.8%. This difference was figured out to be in a direct relationship with the added amount of crocin in the nanoliposome solution. Nevertheless, the amount of nisin did not play a significant role on its encapsulation efficiency (ranged from 44.2% to 49.1%), maybe due to the maximum loading of nisin within nanoliposomes because of the higher attraction of positively charged nisin with negatively charged lecithin present in liposome membranes. A similar result was found in the study of Mohan et al,^29^ investigating the encapsulation efficiency of different charged whey peptides within nanoliposomes.

 Given the findings obtained from particle characterization properties and encapsulation efficiency, the amounts of 4 mg crocin, 4.5 mg nisin, 40 mg lecithin, and the ultrasound time of 40 minutes were selected to synthesis nanoliposomes, so that, this formulation led to the production of nanoliposomes with 199 nm size, 0.3 PDI, -14.6 mV ζ-potential, and 26.2% (crocin) and 48.2% (nisin) encapsulation efficiency.

###  Fourier-transform infrared spectroscopy 

**Figure 1:**
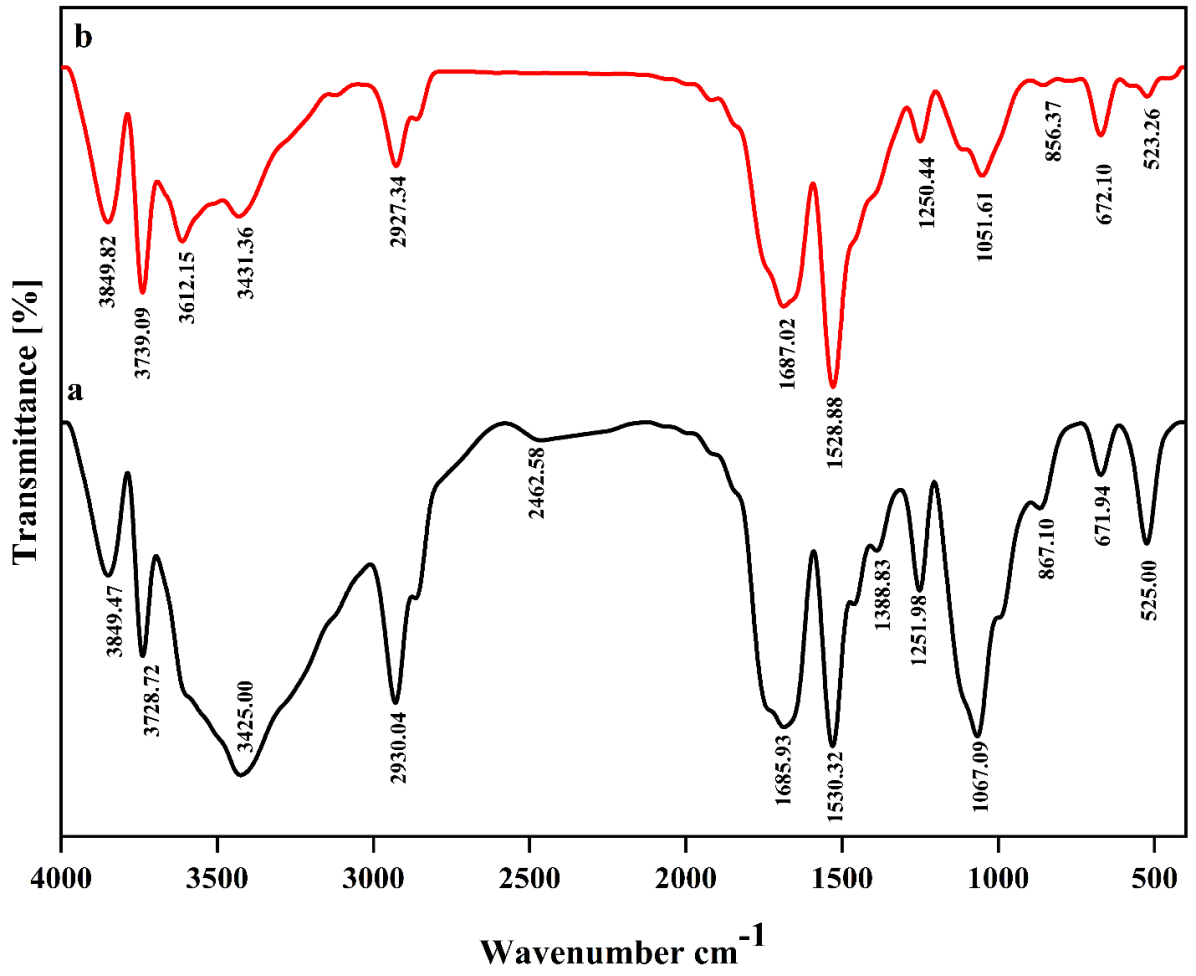
The Fourier Transform Infrared Spectroscopy (FT-IR) spectra of empty nanoliposome (a) and nanoliposome containing both crocin and nisin (b)

 FTIR analysis is commonly used for demonstrating the functional groups of different components in addition to the interactions between functional groups of different substances, showing how they interact with each other. The summary of FTIR analysis in this study showed internal interactions between nanoliposome components along with external interactions between functional groups of bioactive compounds and the nanoliposome components, displaying the formation of prepared nanocarrier system. The FTIR spectra of empty nanoliposome (a) and nanoliposome containing both crocin and nisin (b) are shown in Figure 1. From the unencapsulated spectrum, the carbonyl group (C = O) at 1685 cm ^-1^ and C = C bonds at 1530 cm ^-1^ can be clearly detected. Also, another sign of C = C bonds pertains to the peak bond at 2930 cm ^-1^, verifying the existence of sp2 hybridised carbon. The C = C bond indicates binding between lecithin and cholesterol. The sharp peak at 1067 cm ^-1^ is related to the symmetrical PO^2-^ stretch vibrations of phospholipids, which is close to the absorption peak found in the study of.^30^ The broad range of 3200 cm ^-1^ to 3500 cm ^-1^ is attributed to the presence of OH functional groups of cholesterol structure.

 Given the FTIR spectra obtained from nisin and crocin-loaded nanoliposomes, the most obvious differences could be seen in three absorption regions corresponding to 1067, 1530, and 3200-3500 cm ^-1^ wavelengths. Decreasing the intensity of 1067 cm ^-1^, is probably due to the interactions between phosphate groups of lecithin with carbonyl (C = O) and OH groups of both crocin and nisin.^26^ Also, increasing the intensity of peak at 1530 cm ^-1^ can be justified through the interactions mostly between C-O groups of crocin saccharide part and nanoliposome components, resulting in incrementing of C = C bonds. A huge difference was found in absorption range of 3200-3500 cm ^-1^, and new format of peaks is pertained to the presence of primary and secondary amine stretches in the nanoliposome. As a matter of fact, the interactions between NH groups of nisin and lecithin have probably led to the substitution of OH groups with amine stretches.^31^

###  Antioxidant activity 

 One of the most important aims of nanoencapsulation is to preserve the antioxidant and biological activity of loaded bioactive substances during the process and the storage. In this study, the impact of loading nisin and crocin within the nanoliposomes and nanoliposomes coated with chitosan in a concentration of 1% on their antioxidant characteristics was examined (Table 2). Based on the findings, the lower antioxidant activity (DPPH scavenging activity) was found for samples containing uncoated nanoliposomes containing crocin and nisin (48.23%), which was significantly different (*P* < 0.05) from the samples with free nisin and crocin (59.03%). The percentage of reduction in DPPH absorbance for samples having free bioactives is close to the evaluation result of antioxidant activity of free crocin in the study of Assimopoulou et al,^32^ implying that crocin is the main factor influencing the antioxidant activity of samples. The addition of chitosan to the surface of nanoliposome substantially intensified the reduction in DPPH absorbance (58.0%), and the reason can be attributed to the antioxidant capacity of acetamino (CH_3_CONH-) or amino groups in chitosan.^30^ Overall, it can be concluded that the protection of bioactives in nanoliposomes can be accompanied with desirable results, but it may lead to a decrease in their antioxidant activity.

**Table 2:** DPPH scavenging activity for different formulated milk samples

**DPPH scavenging activity**	**Free crocin and nisin**	**Crocin and nisin-loaded nanoliposome**	**Crocin and nisin-loaded nanoliposome coated with chitosan**
	59.03%	48.23%	58.0%

###  Antibacterial activity

 Maintaining the safety of foodstuff during the storage is the priority of food industries. In this regard, the application of nisin in a number of foods as an antimicrobial agent is a prevalent way to keep the safety of foods. In this study, nisin was expected to control the antibacterial activity of prepared samples, since previous studies have reported the antibacterial activity of nisin against *L. monocytogenes*.^33,34^ Also, crocin was found less effective against *L. monocytogenes *(Data has not been published).

 Antibacterial test revealed that samples with 7.5 mg/L free nisin and 6 mg/L free crocin showed the MIC value against *L. monocytogenes*. However, encapsulated nisin and crocin within nanoliposomes and nanoliposomes coated with chitosan exhibited MIC of 6, 6 mg/L and 4.5, 2 mg/L, respectively, demonstrating higher inhibition activity when the bioactives are encapsulated within nanoliposomes. Similar results were found in the study of.^35,36^ On the other hand, chitosan is a well-known antimicrobial hydrochloride, and its impact against *L. monocytogenes* has been previously proved.^37^ In fact, positively charged amino groups of chitosan interact with negatively charged bacterial cell membranes, resulting in the leakage of proteinaceous and other intracellular compounds of bacteria. Chitosan can likewise bind tracing metals as a chelating agent, thus preventing the formation of toxic substances and the growth of bacteria. Chitosan can act as a water binding material, so that it inhibits the function of various enzymes. Also, owing to the penetration towards the DNA of bacteria and the interference with the production of mRNA, chitosan can decrease the protein synthesizing performance of cells.^38^

###  Color measurement 

 Unwanted colors in foodstuff are normally considered as a negative agent from a visual point of view. One of the most visual expected characteristics of nanoliposomes is to decrease the color intensity of loaded bioactives in food samples.^39^ The effect of adding solutions containing free crocin and nisin, nanoliposome, and nanoliposome coated with chitosan on the L*, a*, and b* values of milk is given in Table 3.

**Table 3:** L*, a*, and b* values of different formulated milk samples

**Milk samples with:**	L*	a*	b*
Free crocin and nisin	-0.91	12.32	8.53
Crocin and nisin-loaded nanoliposome	3.26	5.41	10.92
Crocin and nisin-loaded nanoliposome coated with chitosan	2.85	5.09	10.23

 The result of color measurement analysis exhibited that there were not any significant differences (*P* > 0.05) between milk samples containing both nanoliposome and nanoliposome coated with chitosan in all values of L*, a*, and b*, which demonstrates that chitosan did not play a substantial role in the color of samples. However, samples with free crocin and nisin showed meaningfully differences with other two samples. The most obvious difference was related to a* value, where the sample with the unencapsulated bioactives had a significant higher redness value (12.32) compared to other milk samples. This condition was also seen in values of L* and b*, on the other hand the L* of milk containing unencapsulated crocin and nisin was obtained negative, indicating the lack of whiteness in the samples. Actually, the red color induced by crocin would cause the white color of the milk to disappear. As an overall conclusion, nanoliposome could successfully reduce the redness intensity of crocin in milk samples.

###  Viscosity

 Viscosity is one of the most important characteristics of foodstuff evaluation. Significant changes of viscosity lead to the production of products that are less noticed by the customer. According to the observed findings, the addition of both free bioactives and nanoliposomes without chitosan did not significantly change the viscosity of milk samples. The viscosity obtained for milk containing free nisin and crocin, and milk containing crocin and nisin-loaded nanoliposome was 1.65 cP and 1.71 cP, respectively. However, nanoliposomes coated with chitosan led to an increase in the viscosity of milk samples up to 3.42 cP. Also, the formation of some deposited particles was seen in the chitosan-added samples, which is attributed to the interactions of chitosan with milk proteins, particularly whey proteins.^40^

###  Bacterial growth 

**Figure 2:**
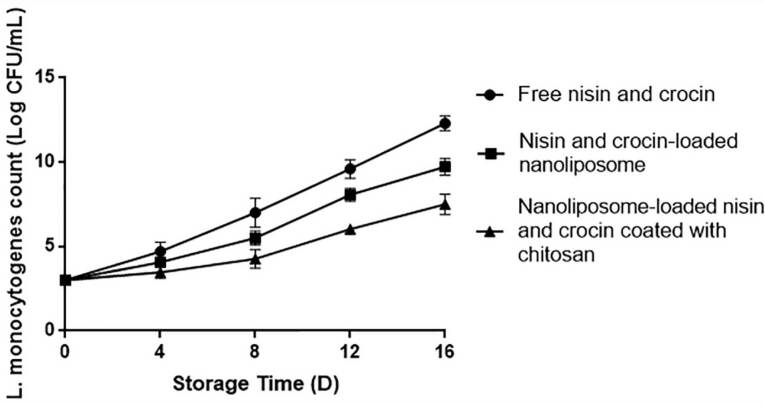
Inhibition on the growth of Listeria monocytogenes during storage days by free nisin and crocin, nisin and crocin-loaded nanoliposome, and nisin and crocin-loaded nanoliposome coated with chitosan

 According to the results obtained from statistical analysis (Figure 2), the highest *L. monocytogenes *count was observed in the samples containing free nisin and crocin (*P* < 0.05) during the days of experiment. The lowest count (*P* < 0.05) was found in the samples with nanoliposomes coated with chitosan. The findings achieved were similar to the antibacterial results obtained in this study, so that the stronger impact of nanoliposomes coated with chitosan against *L.monocytogenes*can be simply attributed to the chitosan antimicrobial features. However, concerning the more influential impact of encapsulated bioactives compared to free bioactives, several reasons have been proposed. The first mechanism is attributed to the passive cellular absorption, meaning that nano size delivery systems, owing to their sub-cellular size, can easily pass antimicrobial substances through cell walls and membranes by the passive cellular absorption mechanism, thereby decreasing the mass transfer resistance.^22^ Also, it is expressed that the encapsulation process within nanoparticles enhances the biological activities of compounds through increasing their concentration and bioavailability because of an increase in the ratio of surface to volume by reducing particle sizes into nano scales.^41^ Besides, nanoliposomes, due to their similarity in the structure to cell membranes, provide successful interactions of encapsulated ingredients with cell membranes. The proposed mechanisms, in which nanoliposomes interact with cells include absorption, fusion, inter-membrane transfer, phagocytosis, and contact release.^42^

## Conclusion

 According to the results, the best formulation was for the samples containing nanoliposomes without chitosan coating. Although, chitosan-coated nanoliposomes showed promising results concerning the bacterial growth and color measurement analysis, the formation of deposited particles along with an increase in the viscosity value are limiting factors of their application in milk. On the other hand, it was shown that the encapsulation of nisin and crocin with uncoated nanoliposomes did not significantly change the viscosity of milk, also, it decreased the color intensity produced by the bioactive compounds and inhibited the growth of *L.monocytogenes*more significantly compared with samples with free nisin and crocin.

## Acknowledgments

 This paper was funded by the Vice Chancellor for Research of Tabriz University of Medical Sciences in Tabriz, Iran (Grant number 5/د/235168, 1397/07/09).

## Author Contributions

**Conceptualization:** Seid Mahdi Jafari.

**Data curation: **Mohammad Yousefi.

**Formal Analysis: **Mohammad Yousefi.

**Funding acquisition: **Ali Ehsani.

**Investigation: **Seid Mahdi Jafari.

**Methodology: **Seid Mahdi Jafari.

**Project administration: **Ali Ehsani.

**Resources: **Ali Ehsani.

**Software: **Hossein Ahangari.

**Supervision: **Ali Ehsani.

**Validation: **Seid Mahdi Jafari.

**Visualization: **Ali Ehsani.

**Writing – original draft: **Mohammad Yousefi.

**Writing – review & editing: **Hossein Ahangari.

## Ethical Issues

 Not applicable.

## Conflict of Interest

 The author has no conflict of interest in this research.

## References

[1] Molayi R, Ehsani A, Yousefi M (2018). The antibacterial effect of whey protein-alginate coating incorporated with the lactoperoxidase system on chicken thigh meat. Food Sci Nutr.

[2] Ehsani A, Paktarmani M, Yousefi M (2017). Efficiency of dietary sodium alginate coating incorporated with lycopene in preserving rainbow trout. Food Sci Biotechnol.

[3] Boor KJ, Wiedmann M, Murphy S, Alcaine S (2017). A 100-year review: microbiology and safety of milk handling. J Dairy Sci.

[4] Flynn BT, Kozak SM, Lawton MR, Alcaine SD (2021). Lactose oxidase: an enzymatic approach to inhibit Listeria monocytogenes in milk. J Dairy Sci.

[5] Drevets DA, Bronze MS (2008). Listeria monocytogenes: epidemiology, human disease, and mechanisms of brain invasion. FEMS Immunol Med Microbiol.

[6] Righeschi C, Bergonzi MC, Isacchi B, Bazzicalupi C, Gratteri P, Bilia AR (2016). Enhanced curcumin permeability by SLN formulation: the PAMPA approach. LWT.

[7] Khatibi SA, Misaghi A, Moosavy MH, Amoabediny G, Akhondzadeh Basti A (2015). Effect of preparation methods on the properties of Zataria multiflora Boiss. essential oil loaded nanoliposomes: characterization of size, encapsulation efficiency and stability. Pharm Sci.

[8] Lu Q, Lu PM, Piao JH, Xu XL, Chen J, Zhu L (2014). Preparation and physicochemical characteristics of an allicin nanoliposome and its release behavior. LWT.

[9] Gibis M, Ruedt C, Weiss J (2016). In vitro release of grape-seed polyphenols encapsulated from uncoated and chitosan-coated liposomes. Food Res Int.

[10] Wang X, Cheng F, Wang X, Feng T, Xia S, Zhang X (2021). Chitosan decoration improves the rapid and long-term antibacterial activities of cinnamaldehyde-loaded liposomes. Int J Biol Macromol.

[11] Gharsallaoui A, Oulahal N, Joly C, Degraeve P (2016). Nisin as a food preservative: part 1: physicochemical properties, antimicrobial activity, and main uses. Crit Rev Food Sci Nutr.

[12] Cerdá-Bernad D, Valero-Cases E, Pastor JJ, Frutos MJ (2022). Saffron bioactives crocin, crocetin and safranal: effect on oxidative stress and mechanisms of action. Crit Rev Food Sci Nutr.

[13] Rahaiee S, Moini S, Hashemi M, Shojaosadati SA (2015). Evaluation of antioxidant activities of bioactive compounds and various extracts obtained from saffron (Crocus sativus L.): a review. J Food Sci Technol.

[14] Rahaiee S, Hashemi M, Moini S, Shojaosadati SA, Razavi SH (2015). Comparison of phytochemical constituents and antioxidant activities of aqueous and alcoholic extracts of saffron. Qual Assur Saf Crops Foods.

[15] Hosseini H, Jafari SM (2020). Introducing nano/microencapsulated bioactive ingredients for extending the shelf-life of food products. Adv Colloid Interface Sci.

[16] Mohamadpour AH, Ayati Z, Parizadeh MR, Rajbai O, Hosseinzadeh H (2013). Safety evaluation of crocin (a constituent of saffron) tablets in healthy volunteers. Iran J Basic Med Sci.

[17] Tavakoli H, Hosseini O, Jafari SM, Katouzian I (2018). Evaluation of physicochemical and antioxidant properties of yogurt enriched by olive leaf phenolics within nanoliposomes. J Agric Food Chem.

[18] Amiri S, Ghanbarzadeh B, Hamishehkar H, Hosein M, Babazadeh A, Adun P (2018). Vitamin E loaded nanoliposomes: effects of gammaoryzanol, polyethylene glycol and lauric acid on physicochemical properties. Colloid Interface Sci Commun.

[19] Hwang JM, Kuo HC, Lin CT, Kao ES (2013). Inhibitory effect of liposome-encapsulated anthocyanin on melanogenesis in human melanocytes. Pharm Biol.

[20] Haghju S, Beigzadeh S, Almasi H, Hamishehkar H (2016). Chitosan films incorporated with nettle (Urtica dioica L.) extract-loaded nanoliposomes: I. Physicochemical characterisation and antimicrobial properties. J Microencapsul.

[21] Huang CM, Chen CH, Pornpattananangkul D, Zhang L, Chan M, Hsieh MF (2011). Eradication of drug resistant Staphylococcus aureus by liposomal oleic acids. Biomaterials.

[22] Wu J, Liu H, Ge S, Wang S, Qin Z, Chen L (2015). The preparation, characterization, antimicrobial stability and in vitro release evaluation of fish gelatin films incorporated with cinnamon essential oil nanoliposomes. Food Hydrocoll.

[23] Zhang J, Froelich A, Michniak-Kohn B (2020). Topical delivery of meloxicam using liposome and microemulsion formulation approaches. Pharmaceutics.

[24] Chi J, Ge J, Yue X, Liang J, Sun Y, Gao X (2019). Preparation of nanoliposomal carriers to improve the stability of anthocyanins. LWT.

[25] da Silva Malheiros P, Sant’Anna V, de Souza Barbosa M, Brandelli A, de Melo Franco BDG (2012). Effect of liposome-encapsulated nisin and bacteriocin-like substance P34 on Listeria monocytogenes growth in Minas frescal cheese. Int J Food Microbiol.

[26] Lopes NA, Pinilla CMB, Brandelli A (2017). Pectin and polygalacturonic acid-coated liposomes as novel delivery system for nisin: preparation, characterization and release behavior. Food Hydrocoll.

[27] Demirci M, Caglar MY, Cakir B, Gülseren İ. Encapsulation by nanoliposomes. In: Jafari SM, ed. Nanoencapsulation Technologies for the Food and Nutraceutical Industries. Academic Press; 2017. p. 74-113. 10.1016/b978-0-12-809436-5.00003-3.

[28] El-Hammadi MM, Arias JL (2019). An update on liposomes in drug delivery: a patent review (2014-2018). Expert OpinTher Pat.

[29] Mohan A, Rajendran SR, Thibodeau J, Bazinet L, Udenigwe CC (2018). Liposome encapsulation of anionic and cationic whey peptides: influence of peptide net charge on properties of the nanovesicles. LWT.

[30] Ramezanzade L, Hosseini SF, Nikkhah M (2017). Biopolymer-coated nanoliposomes as carriers of rainbow trout skin-derived antioxidant peptides. Food Chem.

[31] Niaz T, Shabbir S, Noor T, Rahman A, Bokhari H, Imran M (2018). Potential of polymer stabilized nano-liposomes to enhance antimicrobial activity of nisin Z against foodborne pathogens. LWT.

[32] Assimopoulou AN, Sinakos Z, Papageorgiou VP (2005). Radical scavenging activity of Crocus sativus L. extract and its bioactive constituents. Phytother Res.

[33] Henderson LO, Erazo Flores BJ, Skeens J, Kent D, Murphy SI, Wiedmann M (2020). Nevertheless, she resisted - role of the environment on Listeria monocytogenes sensitivity to nisin treatment in a laboratory cheese model. Front Microbiol.

[34] Churklam W, Chaturongakul S, Ngamwongsatit B, Aunpad R (2020). The mechanisms of action of carvacrol and its synergism with nisin against Listeria monocytogenes on sliced bologna sausage. Food Control.

[35] Ghoochi Atashbeyk D, Khameneh B, Tafaghodi M, Fazly Bazzaz BS (2014). Eradication of methicillin-resistant Staphylococcus aureus infection by nanoliposomes loaded with gentamicin and oleic acid. Pharm Biol.

[36] Khameneh B, Iranshahy M, Ghandadi M, Ghoochi Atashbeyk D, Fazly Bazzaz BS, Iranshahi M (2015). Investigation of the antibacterial activity and efflux pump inhibitory effect of co-loaded piperine and gentamicin nanoliposomes in methicillin-resistant Staphylococcus aureus. Drug Dev Ind Pharm.

[37] Lotfi M, Tajik H, Moradi M, Forough M, Divsalar E, Kuswandi B (2018). Nanostructured chitosan/monolaurin film: preparation, characterization and antimicrobial activity against Listeria monocytogenes on ultrafiltered white cheese. LWT.

[38] Yilmaz Atay H. Antibacterial activity of chitosan-based systems. In: Jana S, Jana S, eds. Functional Chitosan. Singapore: Springer; 2020.p. 457-89. 10.1007/978-981-15-0263-7_15.

[39] Gulzar S, Benjakul S (2020). Characteristics and storage stability of nanoliposomes loaded with shrimp oil as affected by ultrasonication and microfluidization. Food Chem.

[40] Lekshmi RGK, Rahima M, Chatterjee NS, Tejpal CS, Anas KK, Vishnu KV (2019). Chitosan-whey protein as efficient delivery system for squalene: characterization and functional food application. Int J Biol Macromol.

[41] Ribeiro-Santos R, Andrade M, Sanches-Silva A (2017). Application of encapsulated essential oils as antimicrobial agents in food packaging. CurrOpin Food Sci.

[42] Shoji Y, Nakashima H (2004). Nutraceutics and delivery systems. J Drug Target.

